# Cryptococcal meningitis in systemic lupus erythematosus patients: pooled analysis and systematic review

**DOI:** 10.1038/emi.2016.93

**Published:** 2016-09-07

**Authors:** Wenjie Fang, Min Chen, Jia Liu, Ferry Hagen, Abdullah MS, Al-Hatmi, Peilian Zhang, Yun Guo, Teun Boekhout, Danqi Deng, Jianping Xu, Weihua Pan, Wanqing Liao

**Affiliations:** 1 Department of Dermatology, Shanghai Changzheng Hospital, Second Military Medical University, Shanghai 200003, China; 2 Shanghai Key Laboratory of Molecular Medical Mycology, Shanghai Changzheng Hospital, Second Military Medical University, Shanghai 200003, China; 3 CBS-KNAW Fungal Biodiversity Centre, Utrecht 3584, The Netherlands; 4 Department of Medical Microbiology and Infectious Diseases, Canisius-Wilhelmina Hospital, Nijmegen 6532, The Netherlands; 5 Directorate General of Health Services, Ministry of Health, Ibri Hospital, Ibri 515, Oman; 6 Department of Dermatology and Rheumatology, The Second Afflicted Hospital of Kunming Medical University, Kunming, Yunnan Province 650504, China; 7 Department of Biology, McMaster University, Hamilton L8S 4K1, Canada

**Keywords:** cryptococcal meningitis, opportunistic infections, systemic lupus erythematosus

## Abstract

Cryptococcal meningitis is an important fungal infection among systemic lupus erythematosus patients. We conducted a pooled analysis and systematic review to describe the epidemiological and clinical profile of cryptococcal meningitis in systemic lupus erythematosus patients. From two hospitals in China and nine literature databases, cases and prevalence data were collected for pooled analysis and meta-analysis, respectively. Categorical variables of cases were compared using a *χ*^2^-test on the statistical program of SAS. A multiple regression analysis was performed to ascertain independent predictors significantly correlated with prognosis. Meta-analysis was conducted by the statistical program of R. The prevalence of cryptococcal meningitis in systemic lupus erythematosus patients was 0.5%. Patients were predominantly females and adults. A prednisone equivalent of more than 30 mg/day before infection was associated with higher mortality (odds ratio (OR)=9.69 (1.54, 60.73)). In all, 36.8–38.9% patients showed low lupus activity when they developed the crytococcal infection. Moreover, 38.2% of the patients were misdiagnosed. The estimated case-fatality rate was 23.6%. Our results suggest that more emphasis should be placed to further understand lupus-related cryptococcal meningitis and to develop better prophylaxis and management strategies to combat this condition.

## INTRODUCTION

Microbial infections rank as one of the most important causes of morbidity and mortality in patients with systemic lupus erythematosus (SLE), leading to the death of 20%–40% of SLE patients.^1^ The most common risk factors for infection in SLE patients are the use of high-dose corticosteroids, antibiotics exposure,^2^ high SLE activity and intrinsic disorders of cell-mediated immunity.^3^ Moreover, as SLE is a systemic disease, ~20%–70% of SLE patients develop central nervous system (CNS) damage due to autoimmune-mediated attack,^4^ making the CNS susceptible to infection through impairment of the blood–brain barrier.

Cryptococcal meningitis (CM) is a deadly systemic opportunistic fungal infection caused by members of the *Cryptococcus neoformans/C. gattii* species complex, and it primarily occurs in immunosuppressed patients.^5^ Globally, ~1 000 000 HIV-infected patients per year develop CM, and nearly one-third of them die within three months of infection.^6^ The global epidemiological characteristics of CM among non-HIV population remain poorly understood, including the patients with SLE. A recent study suggests that CM is the most frequent mycosis in SLE patients, accounting for 25.8% of invasive fungal infections (IFIs) in these patients^7^ and is ranked as the number one cause of death (85.7%) due to IFIs in SLE patients.^1^ Moreover, *Cryptococcus* ranks as the first or second most important causative agent among the microbial pathogens (including bacteria, fungi and viruses) causing 30.4–58.8% CNS infections in SLE patients.^8, 9, 10, 11, 12^

Because of the unfamiliarity with this infection, the nonspecific clinical presentations at the early stage of the disease of the disease, and the limited information available in the literature, rheumatologists may underestimate the risk of CM and misdiagnose CM as psychosis triggered by steroid treatment,^13^ CNS lupus disease activity^9, 14, 15^ or infection caused by non-fungal pathogens.^16, 17^ The resulting wrong therapy aggravates the infection and delays the administration of the appropriate intervention using antifungal agents.^16, 18, 19, 20^

Dozens of cases and prevalence studies have been reported about CM in SLE patients. The epidemiological and clinical profiles, however, have not been critically reviewed and summarized so far. Thus, we conducted a pooled analysis and systematic review of original and published data. Our objective is to describe the epidemiological and clinical profiles of CM in SLE patients to foster the development of improved prophylaxis and management strategies for this mycosis in SLE patients.

## MATERIALS AND METHODS

### SEARCH STRATEGY AND SELECTION CRITERIA

This study was approved by the Ethics Committee of the Second Military Medical University, Shanghai, China. Given the retrospective nature of the study, the need for informed consent was waived. Our study strictly followed the PRISMA guidelines for reporting systematic reviews, and our protocol was previously registered in PROSPERO (the international database of prospectively registered systematic reviews in health and social care) with registration number NO CRD42015016552. We searched and collected the medical records of all patients who had a confirmed diagnosis of either CM or SLE from January 2001 to July 2015 and were hospitalized at one of in two hospitals affiliated to our university (Shanghai Changzheng Hospital (1280 beds) and Shanghai Changhai Hospital (2000 beds)). This information was used to identify cases of CM among SLE patients. In addition, nine literature databases, namely PubMed, Embase, Web of Science, Wiley Online Library, Springer Link, Science Direct, Cochrane database, Chinese Biomedical Literature Service System (SinoMed) and China National Knowledge Infrastructure, were searched for cases and prevalence studies. The search included all language and publication dates as long as they were archived in the above nine databases. The major search terms used were ‘systemic lupus erythematosus' and ‘cryptococcal meningitis': (‘Lupus Erythematosus, Systemic' [Mesh] OR ‘Lupus' [tiab] OR ‘LE' [tiab] OR ‘Libman Sacks' [tiab]) AND (Cryptococcosis [Mesh] OR ‘Meningitis, Cryptococcal' [Mesh] OR *Cryptococcus* [Mesh] OR ‘*Cryptococcus neoformans*' [Mesh] OR ‘*Cryptococcus gattii*' [Mesh] OR Cryptococc* OR neoformans OR grubii OR gatti* OR Torul*).

This strategy was an example of our retrieval in the database of PubMed, and similar searches have been conducted in the other databases. Articles not written in English or Chinese were professionally translated for further review. The reference lists of the relevant articles were also manually searched to supplement the searches of the computerized databases. All potentially relevant papers were obtained and evaluated in detail. The pooled analysis required extractable information for each case to allow a quantitative analysis. Hence, the authors of related studies were contacted for necessary information if necessary. Cases or case series without detailed medical history and lacking extractable clinical and laboratory data were excluded. Publications were included in the pooled analysis if the criteria for the diagnosis of SLE were followed according to the American College of Rheumatology guidelines,^21, 22, 23^ and the diagnosis of CM was based on the presence of positive findings in at least one of the following tests in cerebral spinal fluid and/or brain tissue samples, including positive *Cryptococcus* culture, India ink staining and cryptococcal antigen (CrAg) test.^24^ Cases without clinical and laboratory data supporting the diagnosis of SLE or CM, even if the authors declared those patients were positively diagnosed, were excluded.

### DATA COLLECTION AND STATISTICAL ANALYSIS

Data from original and published cases were loaded into the EpiData software (version 3.1, The EpiData Association, Odense, Denmark) using a pre-designed form containing the following information: date of inpatient admission, gender, geographic information, age at SLE and CM diagnoses, time from SLE diagnosis to the onset of infection, time from the onset of CM to infection diagnosis, drugs used for SLE control before infection, clinical manifestations and laboratory findings of infection, Systemic Lupus Erythematosus Disease Activity Index (SLEDAI) score at infection diagnosis, information on misdiagnoses, details on antifungal therapies used, and outcome. Corticosteroid doses were converted into prednisone equivalent doses via the online tool available at http://www.globalrph.com/steroid.cgi. For original and published prevalence data, a pre-designed Microsoft Excel form (Microsoft Office, 2010 version, Microsoft Corporation, Redmond, Washington, USA) with the following information was used: continent, country, study base, study design, diagnostic criteria for SLE and CM, study period, number of SLE cases and number of CM–SLE cases. Disagreements were resolved and consensuses were reached through discussion.

SAS (version 9.00, SAS Institute, Cary, NC, USA) was used for the pooled statistical analysis. Results were presented as mean±s.d. for normal data or median with interquartile ranges (IQRs) for non-normal data. The categorical variables were compared using the *χ*^2^-test. A *P*-value less than 0.05 was considered to indicate a statistically significant difference. A multiple regression analysis was performed to ascertain independent predictors significantly correlated with outcome.

The meta-analysis of disease prevalence was conducted using the R (version 3.2.1, The R Foundation for Statistical Computing, Vienna, Austria). Heterogeneity was evaluated using the Q statistic and inconsistency index (*I*^2^) statistic (*I*^2^ values of ~25%, 50% and 75% correspond to low, medium and high heterogeneity, respectively). The pooled data values were generated using the random-effects model. Publication bias was examined by Egger's test (if >10 studies were included in the analysis).

## RESULTS

### STUDIES INCLUDED

For case analysis, a total of 55 cases that met our criteria were identified and extracted. These included five cases from our hospitals and 50 cases from 38 published articles (Supplementary Table S1).

**Figure 1:**
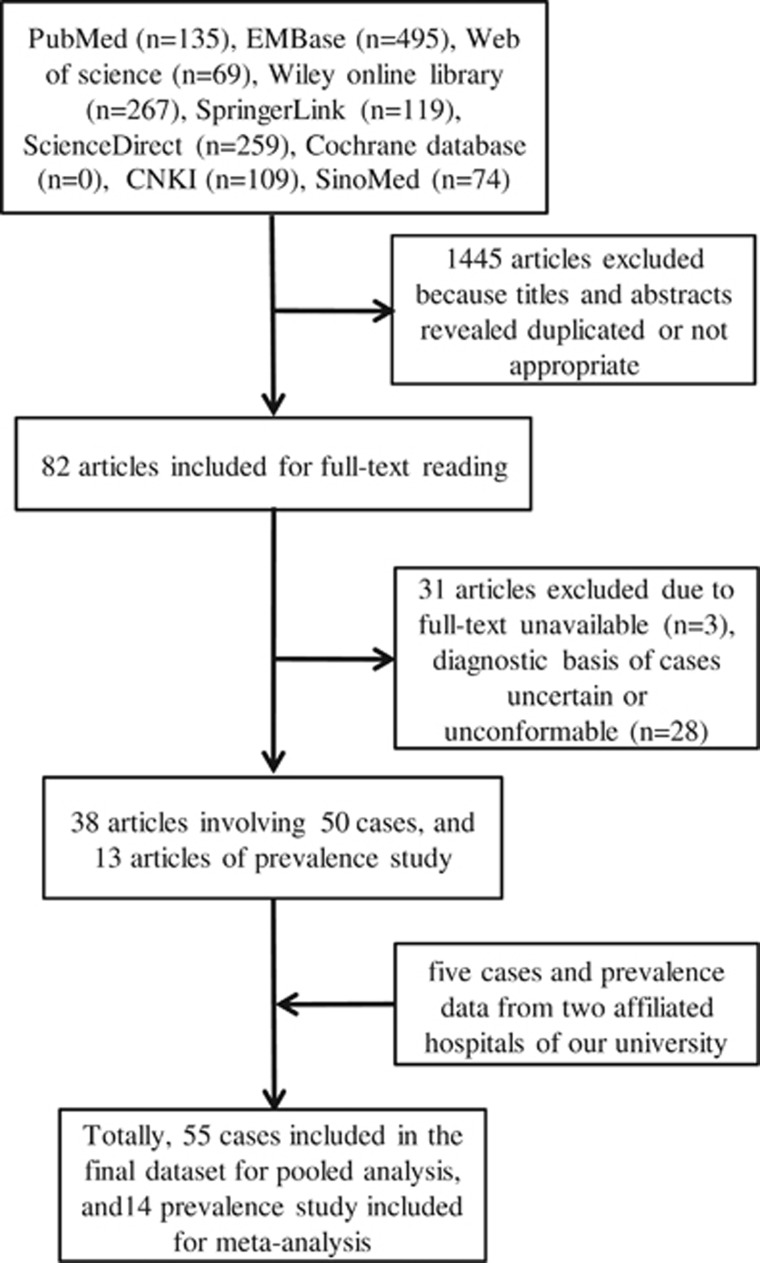
Flow diagram of study selection. China National Knowledge Infrastructure, CNKI; Chinese Biomedical Literature Service System, SinoMed.

For the prevalence meta-analysis, data from 13 studies (Supplementary Table S2) were combined with the original data from two hospitals affiliated to university, resulting in the inclusion of a total of 19 840 SLE cases and 87 CM–SLE cases from 1966 to 2015. Among the 14 studies, five were conducted in the Americas, including Argentina (*n*=1), Colombia (*n*=1), Mexico (*n*=2) and the United States of America (*n*=1), and nine were conducted in Asia, including mainland China (*n*=4), Korea (*n*=1), the Philippines (*n*=1) and Taiwan (*n*=3). Prevalence data reported from Europe, Oceania and Africa were not retrievable. More details on the search strategy used are given in Figure 1.

### EPIDEMIOLOGIC AND DEMOGRAPHIC INFORMATIOn

**Figure 2:**
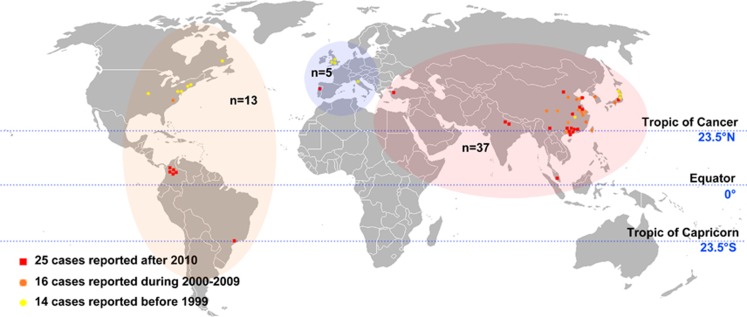
Global distribution of cases. Thirty-seven cases were collected from Asia, 13 from Americas and five from Europe. Fourteen cases were reported before 1999, 16 from 2000 to 2009 and 25 after 2010.

Figure 2 shows the global distribution of the 55 cases of CM–SLE included in the pooled analysis. The patient demographic details are available in Supplementary Table S1. Fourteen cases were reported before 1999, 16 from 2000 to 2009 and 25 after 2010. The majority of cases were collected from Asia (*n*=37; 67.27%), followed by North America (*n*=7; 12.73%), South America (*n*=6; 10.91%) and Europe (*n*=5; 9.09%). No case was reported from Oceania or Africa. More cases were reported from developing countries (*n*=36; 65.5% that is, Brazil, Columbia, China, India, Malaysia and Turkey) than in developed countries (*n*=19; 34.5% that is, the United States of America, Canada, Italy, Japan, Korea, Portugal and United Kingdom).

**Figure 3:**
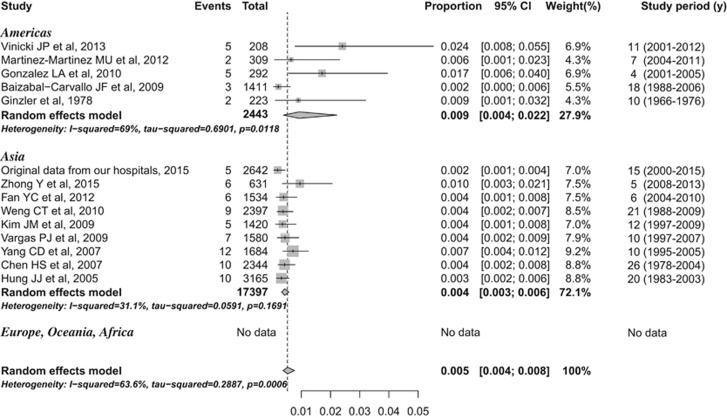
Prevalence of cryptococcal meningitis in systemic lupus erythematosus.

Data from 13 published studies on the prevalence of CM–SLE as well as those unpublished from our hospitals were included in our analyses. The overall pooled prevalence of CM among SLE patients was 0.5% (95% confidence interval (CI), 0.4%–0.8%), and there was a high degree of heterogeneity between studies (*I*^2^=63.6%, *P*=0.001; Figure 3). From the subgroup analysis based on geography, the estimated prevalence was 0.9% (95% CI, 0.4%–2.2%) in the Americas and 0.4% (95% CI, 0.3%–0.6%) in Asia, and this difference was not statistically significant. The levels of heterogeneity in the prevalence of CM among SLE patients in studies conducted in the Americas and Asia were high (*I*^2^=69.0%, *P*=0.012) and low (*I*^2^=31.1%, *P*=0.169), respectively. There was no evidence of publication bias or other small study effects (linear regression test of funnel plot asymmetry, *P*=0.64, Egger's test *P*=0.636). The details on the study design and literature quality assessment are presented in Supplementary Table S2.

The CM–SLE patients were predominantly female (female/male ratio: 8:17). The percentage of cases diagnosed with CM within 1 year of SLE diagnosis was 50.1% (28/55). The median age at definitive CM diagnosis was 32 years (IQR: 21–42 years). The median age at CM diagnosis in developing countries (27 years (IQR: 19–39 years)) was much lower than that in developed countries (40 years (IQR: 25–47 years); *P*=0.03). In contrast, there was no difference in the age at SLE diagnosis between developing and developed countries (*P*=0.10). No patients were found to be HIV-seropositive. Two patients declared being exposed to pigeon droppings, and one patient traveled to a cave region 20 days before experiencing neurological symptoms that constituted an emergency. Before infection, the administration of prednisone equivalents of ≥30 mg/day was associated with higher mortality (*P*=0.02). In addition, more patients received a prednisone equivalent of ≥30 mg/day in developing countries than in developed countries (*P*=0.03), and no patients received a prednisone equivalent of ≥60 mg/day in developed countries. A multiple regression analysis revealed that prednisone equivalent >30 mg (odds ratio (OR)=9.69 (1.54, 60.73)) predicted prognosis of death. However, age of <18 years (OR=0.49 (0.09, 2.67)) and living in developing countries (OR=5.304 (0.86, 32.68)) were not significantly associated with prognosis.

### CLINICAL MANIFESTATIONS AND LABORATORY EXAMINATIONS

The detailed clinical and laboratory findings are available in Supplementary Table S3 (clinical manifestations and laboratory examinations of each individual case) and are partially summarized in Tables 1 and 2. The SLEDAI or SLEDAI-2K score at infection was available in 19 cases. The scores in seven cases (36.8%) were <4 points, and the mean score was 7.58±6.91 points (95% CI, 4.52–10.91). After reviewing the medical charts of the remaining 36 patients whose SLEDAI/SLEDAI-SK scores were not evaluated, 14 (~38.9%) were found to have no indication of SLE activity.

### DIAGNOSTIC PROFILE

To be included in our study, CM infection had to be diagnosed by India ink staining, culture and/or the detection of CrAg. Among the 55 CM cases, India ink staining was positive in 43 patients, negative in seven patients and unknown in the remaining five cases. Cerebral spinal fluid from 49 patients were cultured for evidence of *Cryptococcus* strains; 47 had a positive culture, 39 of which were identified as *C. neoformans* and one of which was identified as *C. laurentii*. CrAg tests were only performed in 20 cases, and one of them was negative. In 11 cases, India ink staining, fungal culture and CrAg tests were positive.

Twenty-one of the fifty-five cases of CM (~38.2%) were initially misdiagnosed. The most common diseases mimicking CM in SLE patients were non-fungal infections, with nine patients being misdiagnosed with bacterial infection (including two with tuberculosis) and two with viral infection. In addition, nine cases were misdiagnosed as CNS lupus and five as lupus activity. Regarding the causes of misdiagnosis, 16 patients did not undergo a diagnostic lumbar puncture (LP) in a timely manner, and the diagnosis of CM in those patients was initially only based on physical examination. Four patients underwent LP but not the CrAg test at initial presentation. The details of the diagnostic profiles are presented in Supplementary Table S4 (diagnostic details of each individual case).

### MANAGEMENT AND OUTCOMES

The estimated case-fatality rate was 23.64% (13/55). Among the 13 patients who died, four (30.8%) died because they abandoned treatment owing to an unaffordable financial burden for antifungal drugs, and one died before receiving treatment. Approximately 38% (21/55) patients received corticosteroids to control active lupus or as an adjunctive therapy to antifungal treatment. The details on the management of CM are listed in Supplementary Table S5 (Therapeutic strategy of each individual case).

## DISCUSSION

CM has a high prevalence and mortality among CNS infections in SLE patients, and is ranked as the most important CNS infection. The prevalence of this disease seems underestimated, and its characteristics have not been adequately described in the literature, causing delay in treatment and control of this lethal invasive infection. Therefore, we conducted a pooled analysis and systematic review of both original and published data to generate a systematic profile of CM in SLE.

In the present study, 55 individual cases were included in the pooled analysis. Despite the fact that CM is normally a male-predominant disease, the patients were mainly females (89.1%), likely because SLE occurs more frequently in females.^25^ This finding indicates that CM may also have a high prevalence in women with SLE, especially in cases with certain female-predominant underlying diseases. More than half (50.1%) of the cases were diagnosed with CM within one year of obtaining a confirmed SLE diagnosis, with a median age at infection of 32 years. However, Wang *et al.*^7^ reported that only 39% of IFIs (including CM) occurred within the first year of SLE diagnosis and that the average age of IFIs was ~35.8±13.5 years. This result indicated that CM tends to emerge relatively early in SLE disease progression compared with other IFIs. Most of the cases were collected from Asia (67.27%), followed by the Americas (23.64%) and Europe (9.09%). The case distribution characteristics were similar to those in a recent systematic review that summarized IFIs in SLE (57.3% cases in Asia, followed by 32.8% in the Americas and 8.7% in Europe, with no data from Oceania or Africa).^7^ However, according to the findings of the current meta-analysis, the CM prevalence in SLE was not higher in Asia than in the Americas. Moreover, there was no difference in SLE prevalence between various continents; for example, the prevalence of SLE ranged from 31.1 to 70.1 cases per 100 000 persons in mainland China, 58.6 cases per 100 000 persons (95% CI, 46.1–73.5) in Argentina and ranged from 14.6–68 cases per 100 000 persons in the United States of America.^26, 27^ This noteworthy inconsistency between the distribution and prevalence of CM–SLE cases could be explained by the fact that the Asian population base (~3.8 billion) is four- to fivefold greater than those in Europe and the Americas (~0.73 and 0.87 billion, respectively). However, the pooled prevalence with high heterogeneity should be interpreted with caution, as it could not be addressed by statistical method. The observed heterogeneity could have come from two main sources. In the first, the included prevalence studies are mostly hospital-based, and the diagnosis of cryptococcosis can vary significantly among hospitals and medical centers. Second, the different epidemiologic characteristics, such as geographically related difference in virulence among strains, the variable genetic susceptibilities to CM–SLE among geographic populations and the different methods of lupus controls among regions, may also have a role. Additional efforts should be made to determine the cause of this prevalence heterogeneity among regions and address the epidemiological profile differences of CM in SLE patients among Africa, Europe and Oceania.

This study revealed that patients taking a prednisone equivalent of ≥30 mg/day before infection had a higher mortality rate. In SLE patients, the CNS was commonly affected (20%–70%).^4^ Damage to the blood–brain barrier accelerated the permeability of corticosteroids, resulting in an increased risk of infection or a worse clinical outcome.^28^ In an SLE population, Staples *et al.*^29^ found that higher prednisone doses were associated with a higher infection rate, however, they did not adequately study the association between prednisone dosage and mortality. One study aimed to explore the relationship between prednisone dosage and mortality from IFIs in SLE.^1^ However, that investigation had a small sample size (*n*=15), and no statistically significant results were obtained. Our study is the first to reveal the relationship between prednisone dosage and mortality from CM in SLE, and this finding may help in directing the research on other IFIs in SLE patients. Our results suggested the importance of effective control of SLE with low dosages of corticosteroids. Further multicenter investigations among cryptococcosis patients with various autoimmune disease conditions need to be designed to address the problem of steroids as an independent risk factor for CM.

The current study highlighted the gap in SLE control between developing and developed countries. Patients in developing countries received higher doses of prednisone and were diagnosed with CM at an earlier stage of SLE than patients in developed countries. SLE patients were more susceptible to infection in developing countries where CM was usually ranked as the leading cause of death.^30, 31^ In developing countries, some medications (even for the basic management of SLE) are not always affordable or available, which has led to the overuse of steroids in place of antimalarial and immunosuppressant drugs and, consequently, a high risk of treatment-related infections. Low therapy adherence is an important risk factor for comorbidities. Low income and education level are associated with poor compliance to therapeutic regimens in lupus.^32, 33^ This phenomenon can be partially explained by the genetic characteristics of the SLE populations in developing countries.^34, 35, 36^ For example, low levels of mannose-binding lectin in Chinese patients with SLE increase the risk of bacterial infection. The management of SLE and prophylaxis for SLE-related infections remains a challenge, and rheumatologists and policymakers should make efforts to establish new management strategies based on the available resources to improve the current situation in developing countries.^37^

The SLEDAI score, a widely used tool for evaluating SLE activity based on clinical manifestation and laboratory tests, was <four points in 36.8% of CM patients. Among the remaining patients in whom the SLEDAI score was not evaluated, 39% exhibited no SLE activity. This percentage is relatively high compared with those reported in previous studies. Among SLE patients with IFIs, only 14% had a SLEDAI<4;^7^ among SLE patients with meningitis 10% had a SLEDAI<4;^10^ and among patients with CNS infection, 4.3%–6%^8^ had a SLEDAI<4.^9^ These results suggested that, even for patients with low SLE activity, the possibility of CM should not be underestimated.

More than one-third (38.2%) of CM cases among SLE patients was misdiagnosed. The most important cause of misdiagnosis is that most physicians (76.19%, 16/21) did not consider CM as a possible diagnosis initially. They made their initial diagnoses based on clinical manifestations or laboratory tests instead of LP followed by cryptococcal tests. CM was generally misdiagnosed as a non-fungal infection, CNS lupus and lupus disease activity. Rheumatologists are commonly unfamiliar with CM and tend to underestimate the prevalence of CM infection among SLE patients. The definitive diagnosis of CM is based on cerebral spinal fluid tests using microscopy (for example, India ink staining), culture and/or CrAg tests after the diagnosis of LP has been made. Although microscopic examination is rapid and inexpensive, it has limited clinical value because of its low sensitivity and specificity. Culture-based methods, as the reference standard for diagnosis, are slow and have a low sensitivity. In contrast, CrAg tests are simple to perform, are less time-consuming, and have a high sensitivity and specificity. In particular, a newly developed CrAg test, namely, the lateral flow immunoassay, was demonstrated to be affordable, sensitive, specific and rapid.^38, 39^ In the reviewed cases, two physicians performed cryptococcal tests other than the CrAg test once the infection emerged, and the CM diagnosis was hampered by the low sensitivity of India ink staining and the time necessary for culture. Therefore, we recommend CrAg tests (especially lateral flow immunoassay) as the routine tests for SLE patients with CNS symptoms and signs.

The management of IFIs, especially CM, is hampered by the unaffordability or unavailability of antifungal agents in resource-limited settings. Notably, our study found that 33% of deaths were attributed to the refusal to take antifungal agents after the establishment of CM diagnosis because the patient could not afford it. In addition, some basic antifungal agents were unavailable in some resource-limited countries. For example, flucytosine is not available in Malaysia. The revised guideline of the Infectious Diseases Society of America^40^ divides CM patients into three treatment categories, namely, HIV-infected individuals, organ transplant recipients and ‘non-HIV-infected, non-transplant' hosts (including SLE patients). The ‘non-HIV-infected, non-transplant hosts' category of CM patients includes both otherwise healthy patients and patients with underlying diseases (for example, autoimmune diseases and cancer). At present, only one set of therapeutic strategy is recommended by this guideline for this category of CM patients despite the heterogeneity within this patient group. In our study, 38.2% patients received corticosteroids to control lupus or as adjunctive therapy to antifungal treatment. However, a recent multicenter randomized controlled trial recommended against the use of corticosteroids with antifungal treatment because of the fact that dexamethasone failed to lower the death rate among HIV-associated CM patients while increasing adverse events (for example, infection, gastrointestinal disorders, renal disorders and cardiac events) compared with a placebo group.^41^ Because SLE control is essential to infection treatment, corticosteroids cannot be simply discontinued. Instead, we believe that the appropriate dosage and timing of corticosteroid use should be further explored. Indeed, more clinical trials (especially randomized controlled trials) should be conducted in the future to facilitate the development of different therapeutic strategies for various populations within the ‘non-HIV-infected, non-transplant hosts' category.

CM is one of the most important CNS infections in SLE patients, with an estimated prevalence of 0.5%. However, CM maybe easily underestimated and misdiagnosed because of the lack of SLE activity and because of the non-standard diagnostic strategy for CM. Emphasis should be placed on prophylaxis for SLE-related infections and management of CM, especially in developing countries where basic agents are unavailable or unaffordable. More efforts, including prevalence studies and clinical trials, are necessary to increase our understanding and improve our control of this disease.

## Figures and Tables

**Table 1:** Clinical manifestations

	**Number**	**Percentage**
Fever	40	72.7
Headache	45	81.8
Nausea	10	18.2
Vomiting	22	40.0
Neck rigidity	17	30.9
Impairment of consciousness	16	29.1
Vision impaired	7	12.7
Papilledema	4	7.3
Seizure	5	9.1
Psychiatric disorder	0	0.0
Twitching	3	5.5

**Table 2:** Laboratory examinations

	**Median**	**Interquartile range**
*Cerebrospinal fluid*		
Intracranial pressure (mmH_2_O)*	260	200, 360
Glucose (mg/L)	37.8	20.16, 50
Protein (mg/L)	1001	500, 1610
Chlorine (mmol/L)	119	112.7, 126
WBC (/μL)	32	4, 85
		
*Blood*		
WBC (10^6^/μL)	6500	5300, 9800
Lymphocyte (10^6^/μL)	725.9	490, 1280
C reactive protein (mg/dL)	17.95	3.5, 99.3
Erythrocyte sedimentation rate (mm/h)	63	34, 102
Complement component 3 (mg/dL)	78.15	58.5, 112.5
Complement component 4 (mg/dL)	11.2	9, 31.6

Abbreviation: White blood cell, WBC.
